# Employing high-frequency alternating magnetic fields for the non-invasive treatment of prosthetic joint infections

**DOI:** 10.1038/s41598-017-07321-6

**Published:** 2017-08-08

**Authors:** Rajiv Chopra, Sumbul Shaikh, Yonatan Chatzinoff, Imalka Munaweera, Bingbing Cheng, Seth M. Daly, Yin Xi, Chenchen Bing, Dennis Burns, David E. Greenberg

**Affiliations:** 1 Department of Radiology, UT Southwestern Medical Center, Dallas, TX USA; 2 Advanced Imaging Research Center, UT Southwestern Medical Center, Dallas, TX USA; 3 Department of Internal Medicine, UT Southwestern Medical Center, Dallas, TX USA; 4 Department of Pathology, UT Southwestern Medical Center, Dallas, TX USA; 5 Department of Microbiology, UT Southwestern Medical Center, Dallas, TX USA

## Abstract

Treatment of prosthetic joint infection (PJI) usually requires surgical replacement of the infected joint and weeks of antibiotic therapy, due to the formation of biofilm. We introduce a non-invasive method for thermal destruction of biofilm on metallic implants using high-frequency (>100 kHz) alternating magnetic fields (AMF). *In vitro* investigations demonstrate a >5-log reduction in bacterial counts after 5 minutes of AMF exposure. Confocal and scanning electron microscopy confirm removal of biofilm matrix components within 1 minute of AMF exposure, and combination studies of antibiotics and AMF demonstrate a 5-log increase in the sensitivity of *Pseudomonas aeruginosa* to ciprofloxacin. Finite element analysis (FEA) simulations demonstrate that intermittent AMF exposures can achieve uniform surface heating of a prosthetic knee joint. *In vivo* studies confirm thermal damage is confined to a localized region (<2 mm) around the implant, and safety can be achieved using acoustic monitoring for the presence of surface boiling. These initial studies support the hypothesis that AMF exposures can eradicate biofilm on metal implants, and may enhance the effectiveness of conventional antibiotics.

## Introduction

Each year in the United States, over one million total knee and hip replacement procedures are performed^1^, and these numbers are projected to increase by 673% and 174%, respectively, by the year 2030 with current population trends and increases in the rates of obesity and diabetes^2–4^. Although PJI only has an incidence of 1–2%^5^, it is one of the most serious complications in the field of arthroplasty. The gold standard in the US for treating PJI is a two-stage revision arthroplasty^6^. In this procedure, the infected implant is removed in a first surgery, the patient is placed on antibiotics for weeks to months to ensure complete eradication of bacteria, then a new prosthesis is implanted via a second surgery^6^. Although this two-stage approach has a cure rate of 90%^7^, it is highly invasive with a significant negative impact on a patient’s quality of life, and is very expensive^4, 8–10^. The projected total annual cost to treat patients with PJIs in the U.S. in 2020 is a staggering $1.6 billion^11^, representing a major healthcare cost.

A major impediment to effective antibiotic treatment of PJI is the presence of biofilm, a thin film (<1 mm^12, 13^) of extracellular polymeric substances (EPS) produced by a microorganism allowing aggregation and adhesion onto the implant surface^14^. This matrix of proteins and polysaccharides shield organisms from the environment^15^, and impairs the effectiveness of both antibiotics and the immune response^16, 17^. Biofilm is associated with infections of many widely used medical implants such as catheters, mechanical heart valves, intrauterine devices, and prosthetic joints^18, 19^. The inability to eradicate biofilm is the main reason replacement of an implant is the standard of care for PJI^19^.

Despite its resistance to antibiotics, biofilm may be eliminated with heat^20, 21^ or other physical means^21–27^. For eukaryotic cells, the cytotoxicity of heat follows a time and temperature relationship, with higher temperatures achieving an equivalent level of cell death in a shorter duration^28^. In addition to killing bacteria, elevated temperatures have been shown to weaken the mechanical integrity of *Staphylococcus epidermis* biofilm^29^. Bacterial eradication with heat is regularly used to achieve surface pasteurization in the food processing industry^30–35^. The principal challenge with applying these thermal methods to infected medical implants is one of access. Most approaches for applying heat or other forms of physical energy require direct surface contact or close proximity to the implant, which is not practical in most clinical contexts. Furthermore, care must be taken to avoid thermal injury to surrounding tissues when considering the application of heat to a prosthetic joint *in situ*.

**Figure 1:**
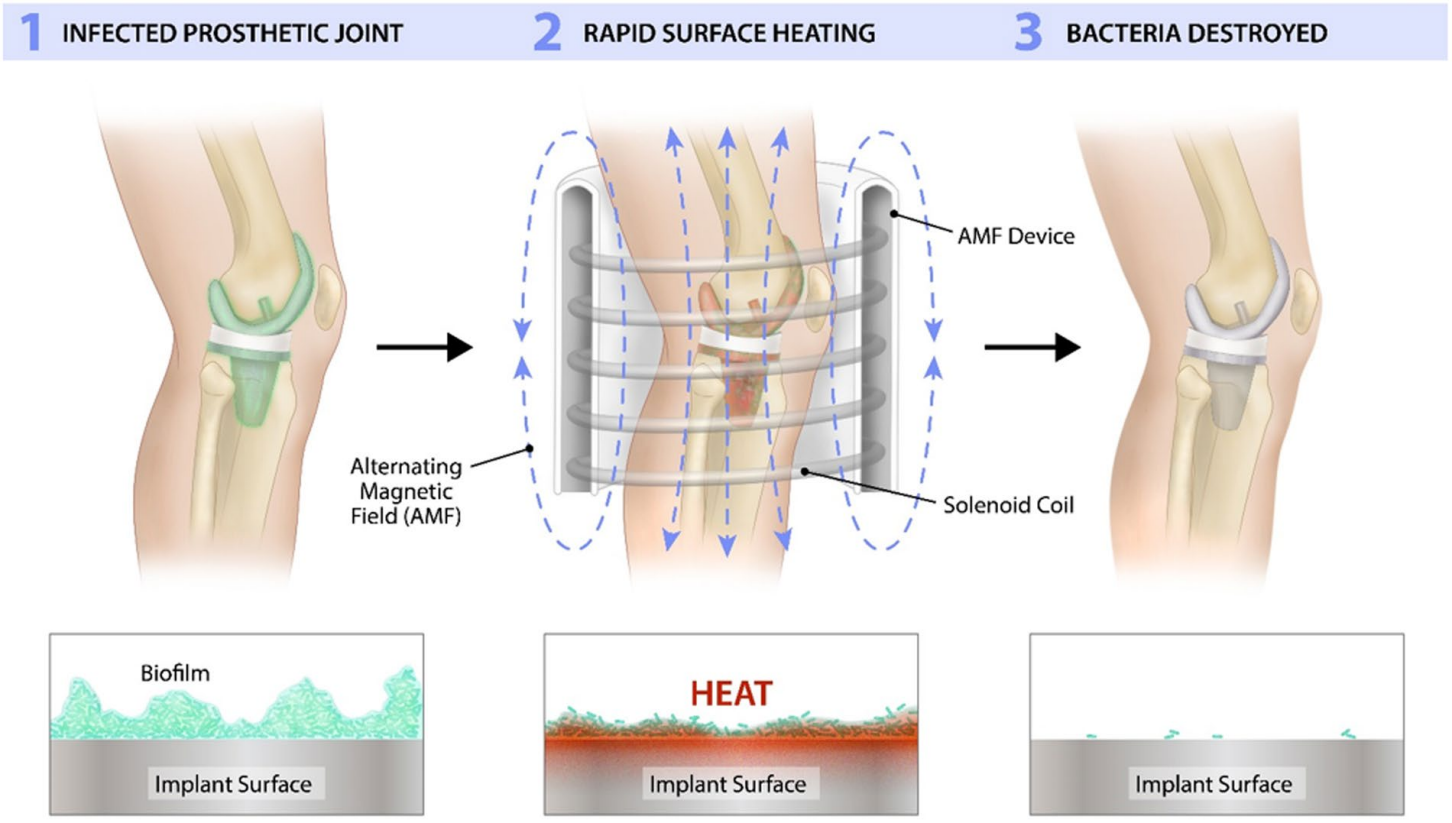
Targeting biofilms located on the surface of metallic implants with high-frequency alternating magnetic fields (AMF). (1) The presence of biofilm on an infected prosthetic usually necessitates surgical replacement as a curative treatment due to an increased resistance of these bacteria to antibiotics. (2) Rapid and non-invasive surface heating of a metallic implant can be achieved using a high-frequency (>100 kHz) alternating magnetic field (AMF). (3) Cytotoxic levels of heating can be generated by the AMF exposures as a means to destroy bacteria and removes extracellular polymeric substances (EPS) associated with the biofilm. This offers a novel and non-invasive method for the treatment of biofilm on prosthetic joints.

Here, we propose a novel non-invasive method for targeting biofilm on metal implants utilizing high frequency alternating magnetic fields (AMF). The concept is depicted graphically in Fig. 1. When metals are exposed to an AMF, electrical currents referred to as eddy currents are induced on their surface. For stainless steel exposed to a 100 kHz AMF, these currents only travel along the outer 1 mm of the object, referred to as the skin depth^36^. Since biofilm is attached to the surface of implants and is less than 1 mm thick, rapid thermal destruction should be achievable while avoiding significant thermal damage to surrounding tissues since only a small fraction of the total volume of a metal implant is heated, and the low electrical conductivity of tissue causes negligible direct heating in these regions. This treatment strategy is only targeted at the biofilm associated with metal implants, but could be used in concert with traditional antimicrobials to treat planktonic bacteria and achieve complete treatment of PJI.

There are precedents for studying AMF exposures as a method for heating implanted metallic objects in applications including tissue hyperthermia^37^, shape-memory alloys^38^, or hyperthermia of intravascular stents^39^. Recently, Coffel and Nuxol^24^ proposed a similar concept in which magnetite nanoparticle composite coatings could be used to render a medical implant responsive to an AMF to treat biofilm with heat, although these coatings may not be required when dealing with bulk metal implants. No prior studies have addressed the issue of safety when considering AMF exposures of metal implants.

The objective of this study is to establish the feasibility and safety of using AMF exposures to eradicate biofilm on metal surfaces, and to assess the interaction of antibiotics and AMF exposures. The extent of thermal damage to surrounding tissues is assessed *in vivo*, and methods to achieve uniform heating of a human prosthetic joint are investigated. The use of remote acoustic monitoring to maintain safe levels of heating during AMF exposures is evaluated *in vitro* and *in vivo*. We hypothesize that surface heating of a prosthetic joint produced by AMF exposures will be bactericidal to pathogens embedded within a biofilm, and will increase their sensitivity to antimicrobial agents. If successful, this completely non-invasive technology would represent a significant advance in the treatment of PJI, avoiding millions of revision surgeries and the corresponding billions of dollars associated with them.

## Results

**Figure 2:**
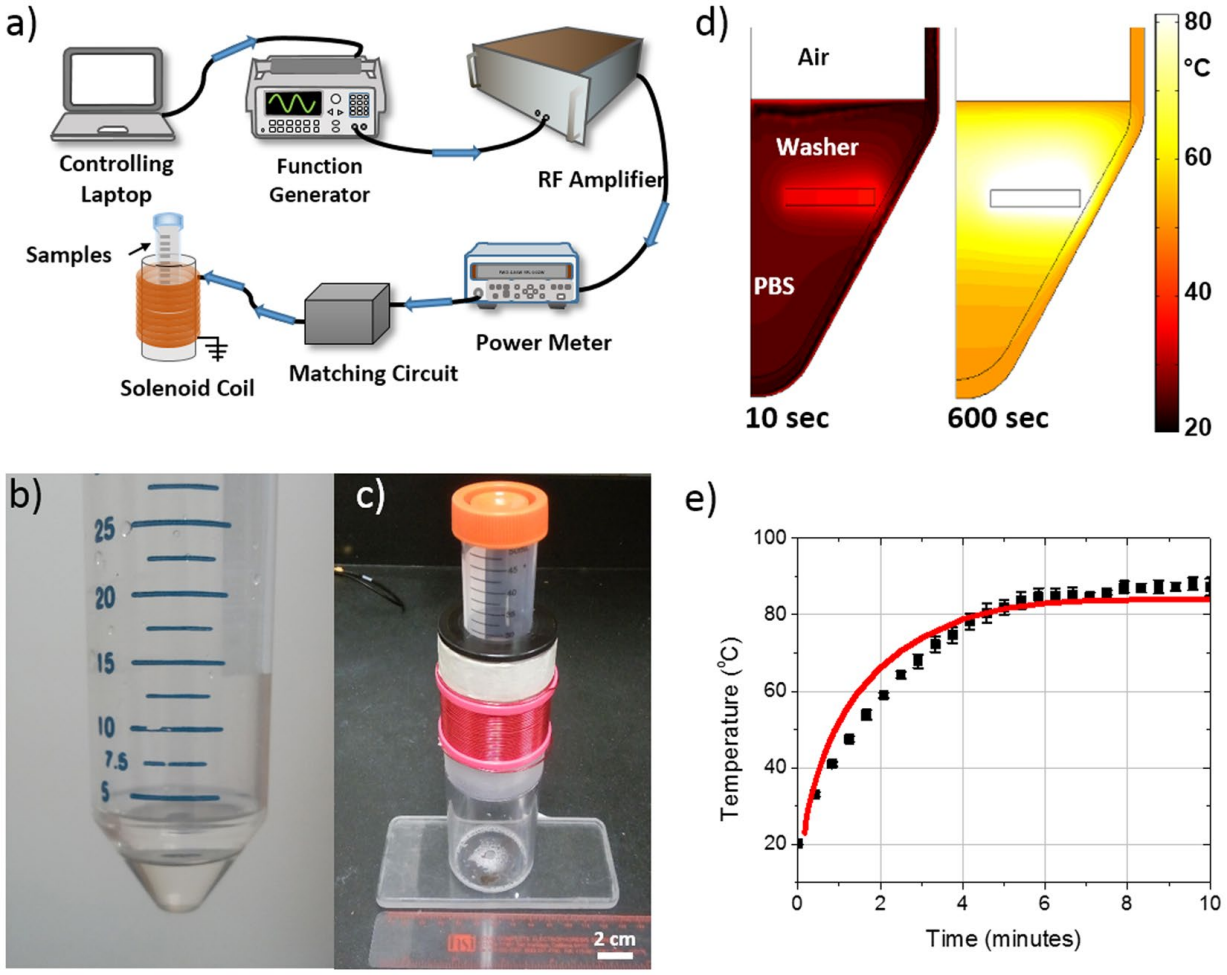
A custom-built solenoid was used to expose metal washers with biofilm to an alternating magnetic field (AMF). (**a**) Under PC control, 20 W was delivered to a solenoid coil at a frequency of 500 kHz. (**b**) Stainless steel washers with biofilm were placed at the bottom of a 50 ml centrifuge tube with 4 ml of PBS. (**c**) The centrifuge tubes were inserted into the center of the solenoid exposed to an AMF for different durations. (**d**) Finite-element simulations depict the spatial temperature distribution inside the centrifuge tube after 10 and 600 seconds of AMF exposure. (**e**) Fiber-optic temperature measurements of the surface of the washer (squares) during a 10 minute AMF exposure show a steady increase in temperature until a steady state value is approached at 6 minutes. The predicted temperature increase from finite-element simulations (red line) and the fiber optic measurements (black squares) agree with a root mean square error of 5.2 °C.

Figure 2a depicts the system used to deliver RF power to a 40-turn solenoid used to produce AMF exposures. A 50 ml centrifuge tube containing 4 ml of PBS and a washer with biofilm was inserted into the center of the solenoid for AMF exposures (Fig. 2b,c). FEA simulations of the interaction between the AMF and the washer (Fig. 2d) confirm the AMF directly heats the washer, and the surrounding PBS is heated indirectly through convection and conduction. Fiber-optic measurements of the washer surface temperature during a 20 W exposure for 10 minutes (Fig. 2e, black squares) demonstrates a steady state temperature of approximately 90 °C (Fig. 2e). The predicted temperatures from the FEA simulations (Fig. 2e, red line) agree with the measured temperatures with a root mean square error of 5.2 °C.

**Figure 3:**
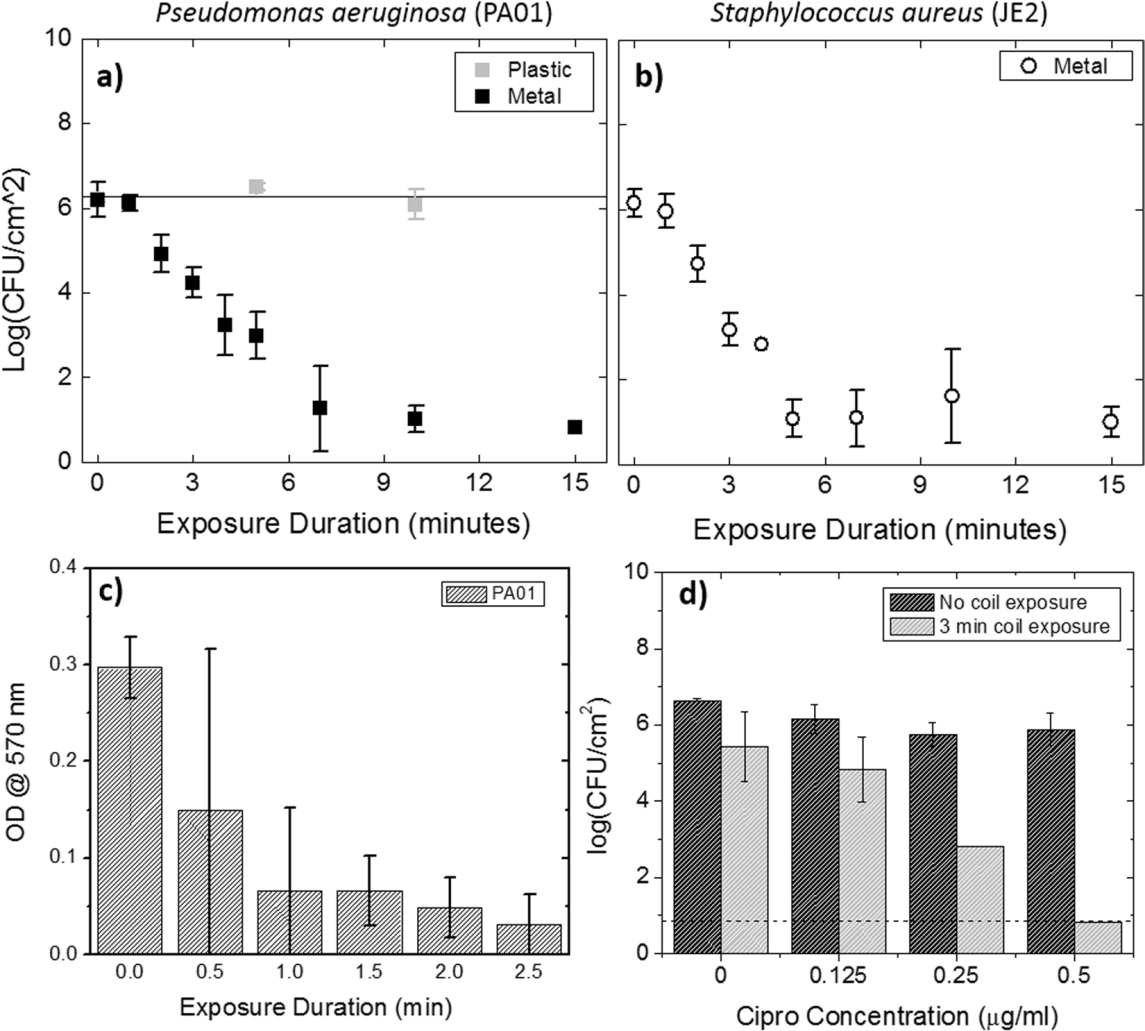
AMF exposures eradicate *P. aeruginosa* and *S. aureus* biofilm and increase their sensitivity to antimicrobials. (**a,b**): The bactericidal effect of a 20 W AMF as a function of exposure duration is shown for two organisms’ biofilm grown on a metal washer. A significant reduction in bacterial number is observed for exposures greater than 1 minute in duration, with a greater than 3-log reduction in the number of bacteria between 5 and 7 minutes (p < 0.0001). The limit of detection (LOD) for the counting assay is shown in each graph as the dashed black line. The results for a control experiment that involved AMF exposures of a similarly sized plastic washer (gray squares in **a**) confirm the effect is due to direct heating of the metal washer. (**c**): Crystal violet staining of metal washers with PA01 biofilm after AMF exposures of increasing duration show a dose-dependent reduction in biofilm matrix, with a significant reduction in staining after 1 minute (p = 0.0037) and the limit of detection reached after approximately 2.5 minutes. (**d**): The ability of an initial 3 minute AMF exposures to dramatically sensitize PA01 biofilm to the antibiotic ciprofloxacin is shown as a function of the concentration of ciprofloxacin. At 0.5 µg/ml, a 5.0 ± 0.4 log reduction in bacterial number was observed for biofilm which received an initial 3 minute AMF exposure (p < 0.0001). Without the AMF, there was no significant reduction in the number of bacteria at all concentrations (p > 0.1). All results shown in the graphs are biological triplicates and are shown as mean and standard deviation.

Figure 3 shows the reduction in colony forming units (CFU – log scale) as a function of AMF exposure duration for two biofilm-producing organisms: (a) *Pseudomonas aeruginosa* (PA01), and (b) *Staphyloccocus aureus* (JE2). For both organisms, there was a statistically significant reduction in CFU observed after 2 minutes (p < 0.01), and a greater than 3-log reduction (p < 0.0001) observed after 5 and 7 minutes for *P. aeruginosa* and *S. aureus* respectively. Beyond 7 minutes, the limit of detection for the assay (1 × 10^1^ CFU/cm²) was reached for both strains representing a > 5-log reduction. The observed trends were very similar for both PA01 and JE2, suggesting that the mechanism of bacterial killing is a direct physical effect of the heat produced by the AMF. Control experiments in which PA01 biofilm was grown on a plastic washer demonstrated no CFU reduction with AMF exposures up to 10 minutes in duration (Fig. 3a – light gray squares).

Figure 3c depicts the early changes in the matrix of a PA01 biofilm exposed to AMF utilizing a crystal violet assay^40^. A significant reduction in the amount of crystal violet, measured as the optical density of a stained biofilm, is observed within 1 minute (p = 0.0037), and the readings reach the limit of detection for this assay by 2.5 minutes. The impact of these early changes in the biofilm matrix on the sensitivity to antimicrobials is shown in Fig. 3d. Metal washers with an 18 h PA01 biofilm on their surface were incubated with increasing sub-inhibitory concentrations of ciprofloxacin for an additional 18 hours before sonication and CFU measurement. One group of washers was incubated with antibiotic alone, whereas another group received a 3 minute AMF exposure prior to incubation with ciprofloxacin. The results, shown in Fig. 3d, demonstrate a dramatic increase in the sensitivity of PA01 biofilm to ciprofloxacin after an initial 3-minute AMF exposure. In the absence of AMF, the reduction in CFU with increasing ciprofloxacin concentration was not significant for all concentrations (all pair-wise p values > 0.1), as expected for a biofilm since 0.5 μg/ml represents the MIC for planktonic PA01. Exposing the washers to a 3-minute 20 W AMF without any antibiotics had a minor, but statistically significant CFU reduction (p = 0.02). However, the application of a 3-minute AMF exposure prior to incubation with 0.5 μg/ml ciprofloxacin resulted in a mean reduction of 5.1 ± 0.4 Log (CFU/cm^2^) (p < 0.0001) relative to the group with no AMF exposure. The effect of the combined treatment was greater than each alone, suggesting a synergistic effect of AMF exposures and ciprofloxacin.

**Figure 4:**
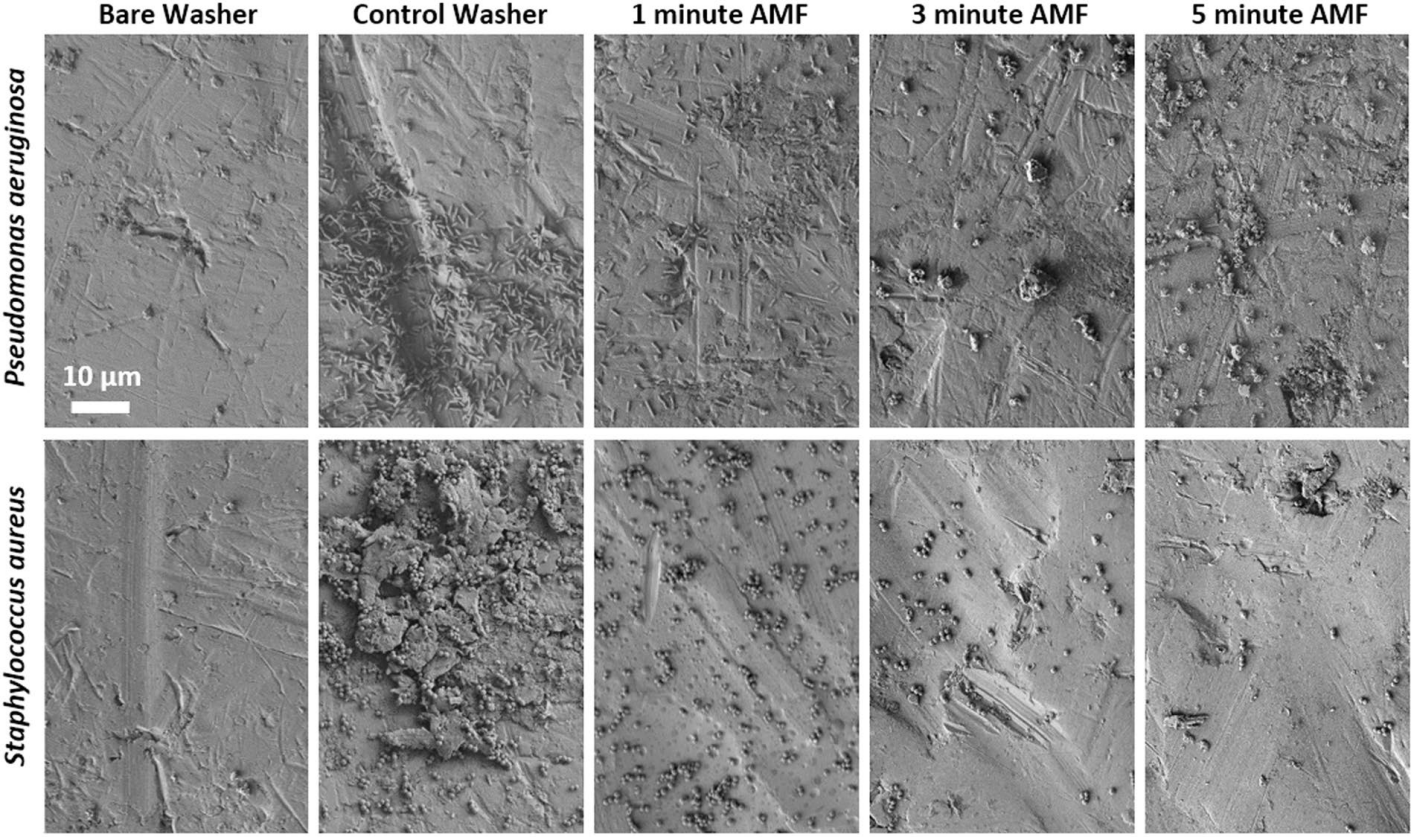
Scanning electron microscopy reveals that AMF exposures remove both EPS and bacteria found within biofilm. The two rows show the results for different bacteria (top – PA01, bottom – JE2) and the columns show images acquired for washers exposed to different durations of AMF. A negative control (washer with no bacteria) is shown in the left most column, and a positive control (washer with biofilm but no AMF exposure) is shown in the second column. The EPS appears to be removed after 1 minute of AMF exposure, although bacteria are still visible on the washer surface. For exposures greater than 3 minutes, it was difficult to find any bacteria on the washers, and only scattered aggregates of material were visible on the washer surface.

**Figure 5:**
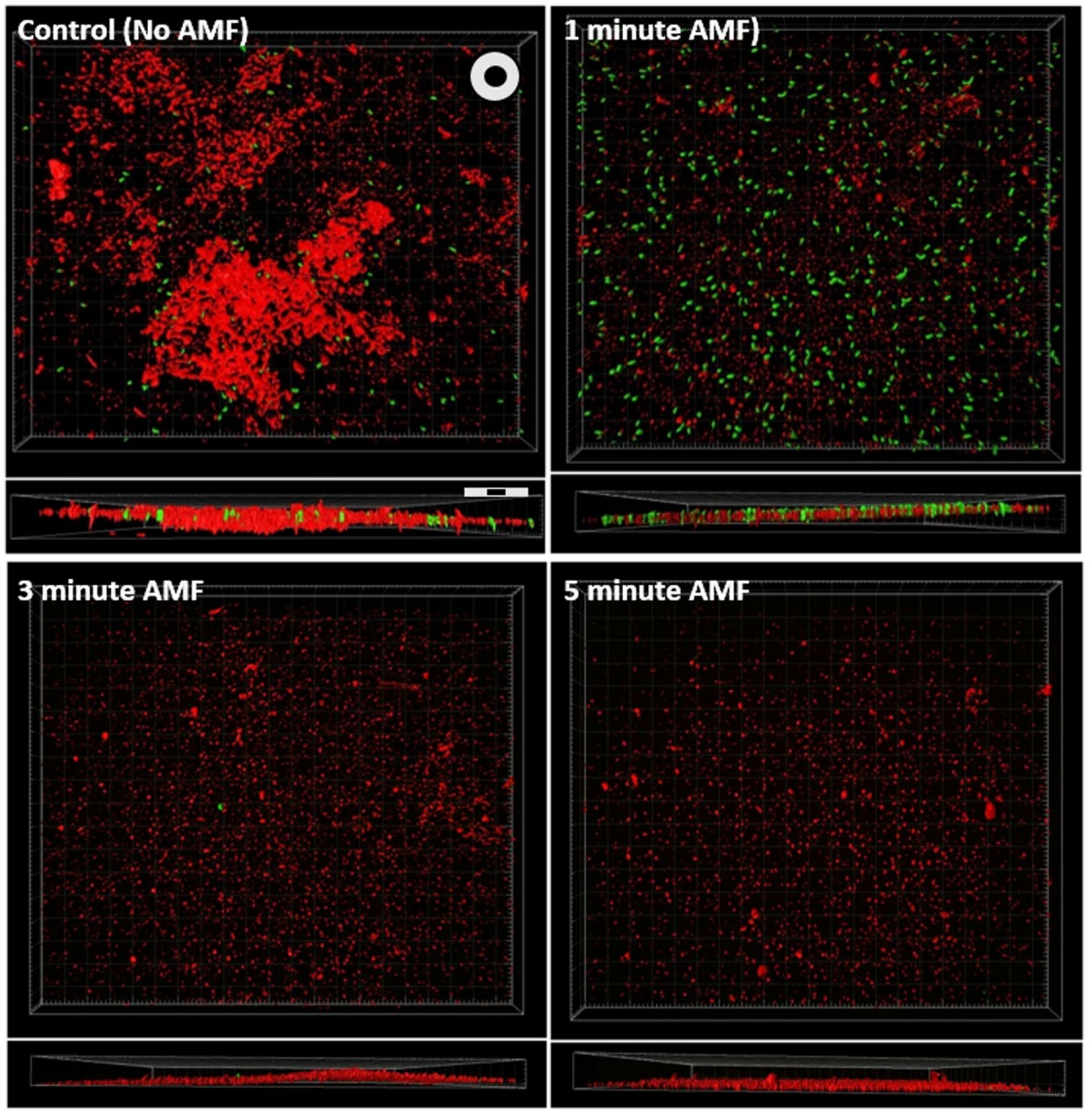
Confocal microscopy of GFP expressing *P aeruginosa* biofilm on a metal washer support SEM findings that AMF initially targets EPS. Confocal microscopy images of infected washers exposed to AMF show a decrease in the biofilm matrix (red) and number of bacteria (green) as the duration of an AMF exposure was increased. For exposures greater than 3 minutes it was difficult to detect any bacteria on the washer surface. At 1 minute, the number of bacteria appear relatively unaffected, but the biofilm matrix signal is greatly decreased, suggesting heating initially affects this matrix. The washers were incubated in GFP-PA01 for 18 hours to form a biofilm. The scale bars in each panel indicate 20 μm.

A deeper understanding into the mechanism of AMF cytotoxicity was obtained with scanning electron and confocal fluorescence microscopy. Figure 4 shows the appearance of PA01 (top row) and JE2 (bottom row) biofilm before and after AMF exposure. A control washer (no AMF) shows the presence of bacteria across the metal surface as well as matrix EPS components of the biofilm. Some features are related to the surface roughness of the washer, as seen in the bare washer (no biofilm) images. After a 1 minute AMF exposure, the matrix EPS component of the biofilm appears to have been removed, even though the number of bacteria appear relatively unchanged. After 3 and 5 minute AMF exposures, there is a time-dependent reduction in the number of bacteria, similar to the observed CFU reduction. In the case of PA01, there remain scattered aggregates of material across the surface of the washer that may be remnants of the EPS, although these were not present in the case of JE2. Although the EPS matrix is difficult to see in the SEM images for PA01, the findings are supported by confocal fluorescence microscopy of washers with GFP-expressing PA01 biofilm. As depicted in Fig. 5, a control washer with biofilm shows the matrix EPS in the red channel, and GFP-expressing PAO1 bacteria in the green channel. The thickness of the biofilm is also shown for each case. After a 1 minute AMF exposure, the biofilm matrix components are largely gone, however, the individual bacteria are still visible on the surface of the washer. After 3 and 5 minute exposures both the EPS and bacteria are significantly reduced. Taken together, the microscopy results and crystal violet staining strongly suggest that the early impact of AMF exposures on these metal washers is to remove the biofilm EPS matrix.

**Figure 6:**
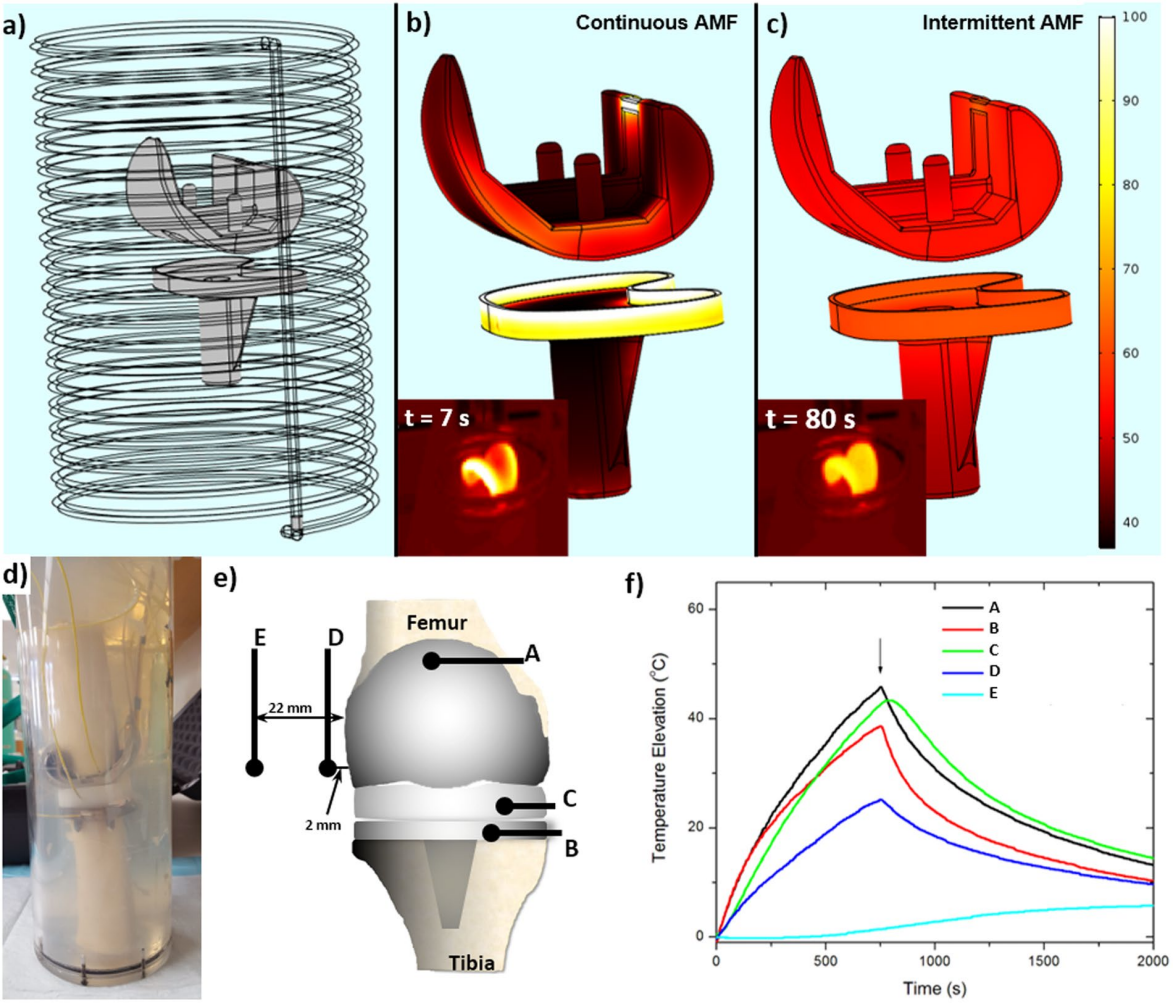
FEA modeling and experiments demonstrate an intermittent AMF exposure can achieve uniform heating across the surface of a prosthetic knee implant. (**a**) Using COMSOL® a 3D representation of a prosthetic knee implant inside a solenoid coil can be defined. (**b**) The surface temperature distribution after 3 seconds of continuous 600W AMF exposure is very non-uniform (over 60 °C range across surface). (**c**) Using an intermittent AMF exposure (1500W for 1 second, every 50 seconds) a uniform surface temperature distribution is achieved after 1200 seconds (20 exposures). The infrared images of the femoral component of a knee prosthesis exposed to AMF (insets of b) and c)) depict the different surface temperature distributions between continuous and intermittent exposures. (**d**) A prosthetic knee implant was embedded in a phantom composed of bovine bone and muscle-equivalent gel material. (**e**) Fiber-optic temperature sensors were embedded in the phantom at the locations shown in the schematic. (**f**) Temperatures recorded over time during an intermittent AMF exposure (300 ms in duration, applied every 15 seconds) demonstrate that after 10 minutes, the top, middle and bottom surfaces were within a few degrees of each other, and were elevated approximately 40 °C above baseline. Further, while the tissue 2mm from the implant is elevated approximately 20 °C above baseline, tissues 22 mm from the implant only experience a temperature elevation of a few degrees C. Movies of the simulations in b) and c) are included as supplemental files.

In addition to evaluating the efficacy of AMF exposures *in vitro* on biofilm, the feasibility of translating this treatment strategy to a human-sized implant was investigated through numerical simulations and phantom studies. 3D CAD models of a prosthetic knee joint and solenoid coil were created and imported into the FEA software package COMSOL®, as shown in Fig. 6a. The surface temperature distribution on the prosthesis produced by AMF exposures from the solenoid was calculated for different combinations of power, duration and delays. As depicted in Fig. 6b, the surface temperature after a single 600 W AMF exposure 3 s in duration is very non-uniform, ranging from approximately 40 to 100 °C. This heating distribution is undesirable because soft tissue near the high temperature regions are at risk of thermal damage while sections of the prosthesis surface remain below temperatures necessary to eradicate biofilm. To address this challenge, an intermittent strategy was explored, in which short duration AMF exposures are applied, followed by a delay. The greater thermal conductivity of metals as compared to tissue facilitates preferential heat transfer along the prosthesis from high to low temperature regions. An example of the spatial temperature distribution achievable with this type of intermittent AMF exposure is shown in Fig. 6c, where twenty AMF exposures were delivered, each with a transmitted power of 1500 W for 1 second, and a 50 second delay in between. The surface temperature distribution after 20 exposures is much more uniform than Fig. 6b, ranging between 50 and 65 °C. Infrared imaging (Fig. 6b,c insets) of a prosthetic knee joint (in air) exposed to a continuous or intermittent AMF from a 2 turn solenoid support the simulation predictions. The non-uniform surface temperature after a 7 second continuous AMF exposure at 1500 W is evident, as is the more uniform distribution achieved with an intermittent approach in which the prosthetic received a 300 ms AMF exposure at 4000 W every 15 seconds. The differences in the AMF powers and delays between the simulations and experiments are due to the different solenoid configurations, and the fact that the prosthesis was in air for the infrared measurements versus tissue for the simulations. The higher power applied compared to the washer are due to the overall increase in the size of implant and the increased diameter of the coil, plus a desire to reach therapeutic temperatures in a shorter time to minimize thermal damage to surrounding tissues.

Figure 6d shows a photograph of a joint phantom constructed using a human prosthetic knee implant and bovine bone, embedded in muscle-equivalent gel material^41^. Fiber-optic temperature sensors were embedded in the prosthetic joint at locations shown in Fig. 6e. As seen in Fig. 6f, the temperature at all locations on the joint surface (A–C) increased at a relatively uniform rate using an intermittent AMF exposure (300 ms AMF, 1500 W, 15 second delay). The sensor on the joint surface was located at a point on the surface of the implant that had a lower temperature elevation, and the maximum temperatures on the implant were likely up to 40 °C higher, especially close to the sensors embedded in the gel. The first such sensor located 2 mm from the joint surface also increased steadily with time but at a slightly lower rate than the joint surface, whereas the sensor located 22.5 mm away did not heat significantly until well after the power was turned off (arrow in Fig. 6f). The total temperature elevation in this experiment may be greater than what is necessary *in vivo* if antimicrobials are also used.

**Figure 7:**
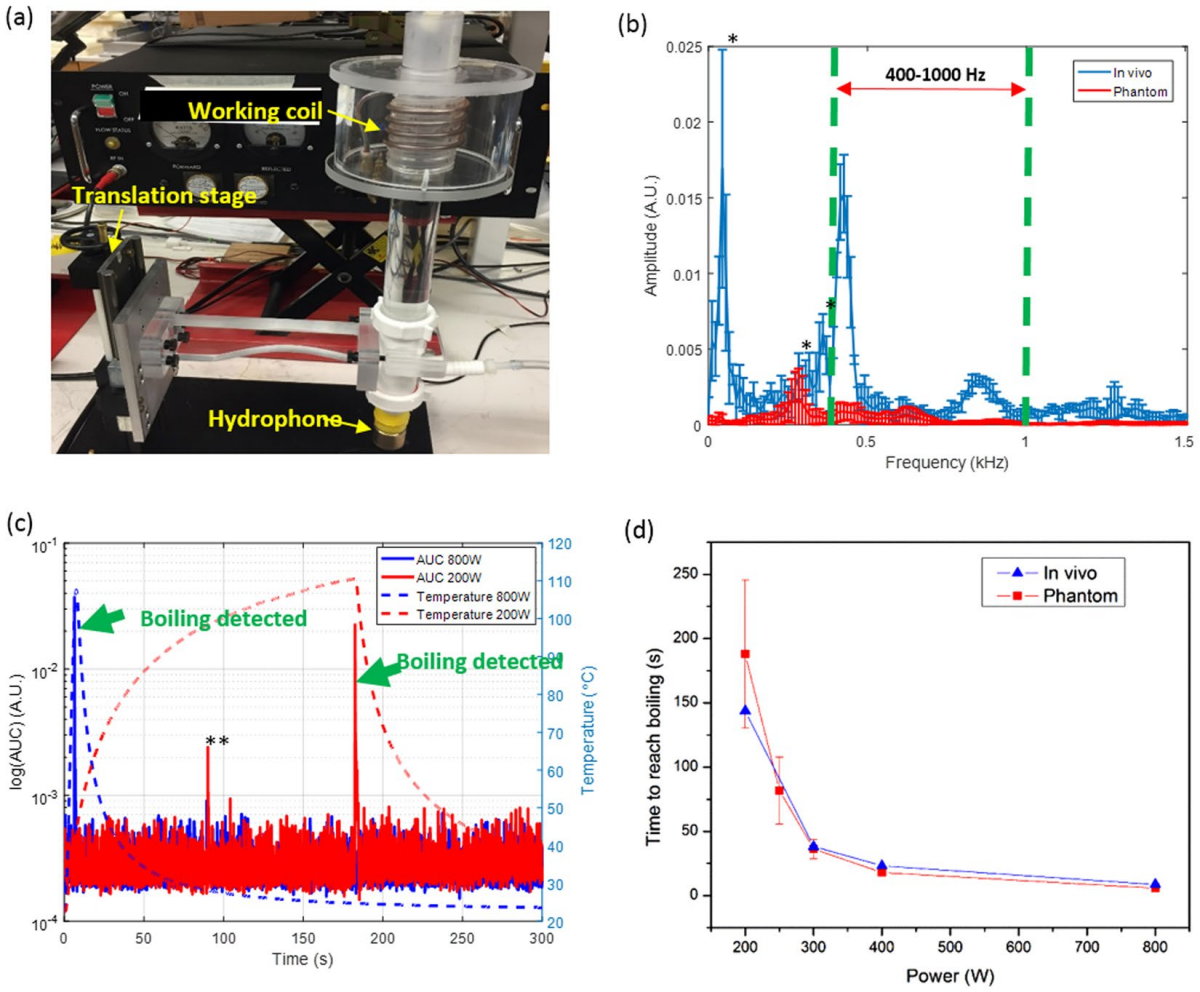
Boiling detection for AMF safety control. (**a**) The AMF system with boiling detection, including: working coil, hydrophone, and translation stage; (**b**) Frequency spectrum of the sound signals acquired from the hydrophone when boiling occurs both *in vivo* (n = 4) and in tissue-mimicking phantoms (n = 4); *: environmental noise; (**c**) Relationship between AUC and temperature of the metal implant in tissue-mimic phantoms. Both high power (800W, blue curves) and low power (200W, red curves) exposure were shown. **: a wrench fell down on the table; (**d**) Relationship between boiling time and exposure power both *in vivo* and in phantom.

A natural question that arises with this proposed concept is that of safety to surrounding tissues. If any location on the metal implant exposed to AMF exceeds 100 °C, boiling will occur immediately adjacent to the implant surface. The formation and collapse of bubbles associated with boiling should produce acoustic emissions detectable remotely using an appropriate receiver, within microseconds of their onset due to the speed of sound in tissue (1500 m/s). This acoustic signal could serve as a form of wireless temperature detection, and could be used to turn off or modulate AMF delivery. The feasibility of this remote acoustic detection was evaluated in phantom and mouse experiments. Figure 7a shows the experimental setup which consisted of a custom made 4-turn solenoid, and an acoustic hydrophone connected to the coil center by a column of water for sound transmission. The *in vitro* experiments involved exposing a 4.8 mm stainless steel ball embedded in a tissue-mimicking phantom^41^. For the *in vivo* experiments, the metal ball was implanted into the dorsal thigh muscle of a mouse. In both cases, the embedded ball was located at the center of the solenoid coil and exposed to AMF. Figure 7b shows the frequency spectrum measured with the hydrophone when boiling was detected in the phantom and *in vivo*. An increase in the amplitude across multiple frequencies ranging from 0 to 1500 Hz is observed, with a consistent increase between 400 and 1000 Hz across multiple experiments. An area under the curve (AUC) was calculated by integrating the amplitude of the frequency spectrum between 400 and 1000 Hz. Figure 7c shows the relationship between this AUC and temperature for the phantom. The increase in AUC when boiling is present is over 2 orders of magnitude over the baseline value of the AUC. Further, simultaneous fiberoptic temperature measurements confirm that the increase in the AUC occurs when the temperature of the ball reaches approximately 105–110 °C for either fast or slow heating. The only likely physical explanation for why boiling appears to occur above 100 °C is the placement of the optical fibers in the center rather than surface of the ball. Finally, Fig. 7d shows the time required to reach boiling on the surface of the ball for the *in vitro* and *in vivo* models. Above 400 W, boiling is achieved in a matter of seconds, reinforcing the ability to generate rapid surface heating with AMF exposures.

**Figure 8:**
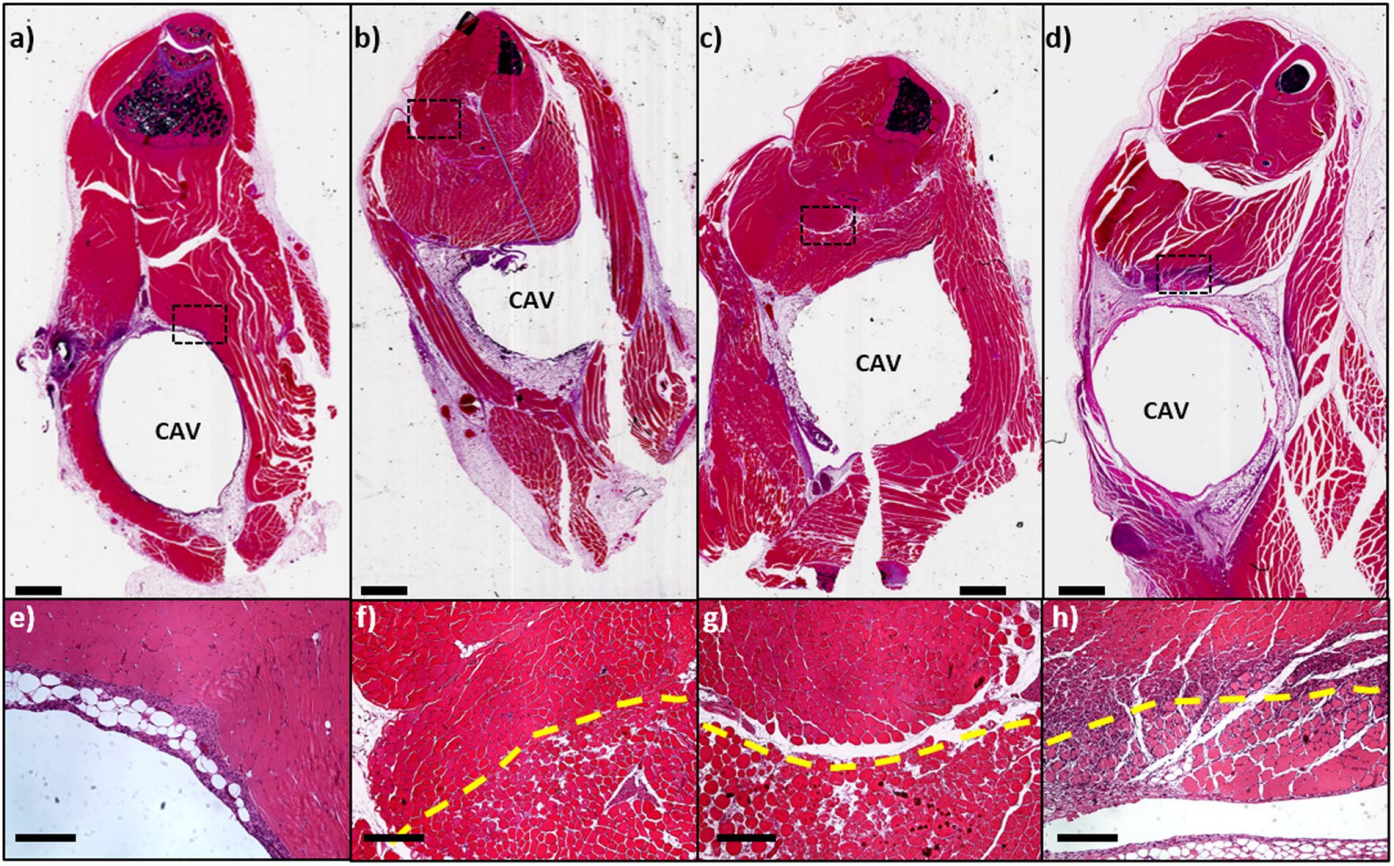
AMF exposures produce highly localized tissue damage *in vivo*. H&E stained sections through the thighs of mice receiving AMF exposures. The cavity (CAV) represents the location of a surgically implanted 5 mm stainless steel ball. The bottom panels are high power views of the ROI indicated in each of the top panels. A sham-treated mouse (**a**,**e**) 7 days after surgical implantation shows a residual mild inflammatory response at the edge of the cavity. A mouse receiving a 190 W AMF exposure for 220 s (**b**,**e**) exhibits a pattern of thermal damage all around the implant approximately 2 mm from the cavity. A similar pattern of damage is seen for a mouse exposed to 800 W for 15 seconds (**c**,**g**), however the radial extent is only approximately 1 mm. A mouse 7 days after an 800 W AMF exposure lasting 4 seconds (**d**,**h**) exhibits a circumferential pattern of damage extending to approximately 0.5 mm from the cavity rim. In addition, robust regenerative activity is seen at the edge of the thermal damage boundary. In all cases, the transition from damaged to normal muscle (dashed line in bottom panels) is abrupt. The top scale bars represent 1 mm and the bottom bars represent 200 µm.

The ability to detect acoustic emissions associated with boiling was confirmed in all mouse exposures. The strength of the acoustic emissions was greater than for the balls embedded in tissue-mimicking gel, presumably due to the higher water content around the ball in the muscle. Figure 8 shows H&E stained sections through the muscle at the location of the implanted ball (represented by a cavity in the images), where top panels are full sections and bottom panels are higher magnification (40x) views of the regions indicated by the dashed boxes in the top panels. Control animals exhibited a robust inflammatory response from the surgical implantation, which was confined to the implant-tissue boundary at 7 days (Fig. 8a,e), and largely gone after 14 days (data not shown). Figure 8b,f and c,g show tissue sections through muscle exposed to a low and high power AMF respectively, while Fig. 8c,g and d,h show the acute and 7 day response to a high power AMF exposure. It is evident that increasing power and decreasing exposure time, resulted in a reduced radius of thermal damage around the cavity. Within the region of thermal damage contraction bands were observed in muscle fibers, connective tissue exhibited a homogeneous appearance, and vascular channels were distended with coagulated blood contents. Further, there was a greater level of fragmentation of muscle fibers and separation between myocytes related to expansion of the endomysium. Seven days after the AMF exposure, myocytes within the region of thermal damage exhibited evidence of irreversible injury in the form of coagulated, necrotic sarcoplasm. The boundary of thermal damage was well defined, with significant regenerating myofibers at the periphery making incursions into the region of thermal damage. Evidence of repair was evident at the periphery of the necrotic zone in the form of local fibroblastic proliferation and macrophage infiltration.

## Discussion

In this study, we confirm the hypothesis that non-invasive heating by AMF produced on metals bactericidal. Furthermore, based on SEM and confocal imaging, the initial effect of these AMF exposures appears to be removal of the biofilm EPS rendering bacteria much more sensitive to antimicrobial agents. This observation has important clinical implications with respect to consideration of combined AMF and antimicrobial strategies, but also for the potential to lower thermal exposures to reach a desired therapeutic endpoint.

Preserving soft tissues and structures surrounding the target implant from thermal damage is a key safety requirement for this technology. Wireless detection of temperature can be realized using acoustic emissions when surface boiling is present on the implant as a means of monitoring for unsafe conditions. Tissue thermal damage is time and temperature dependent, therefore even temperatures below boiling could result in damage if soft tissue is exposed for long periods. *In vivo* experiments confirmed that tissue damage can be confined to within a few mm of the implant, with a tendency for reduced distance with higher power and shorter duration exposures. Finally, the impact of AMF on the integrity of implant-bone adhesion is also an important factor that needs further study, as well as the influence of different surface characteristics of implants on AMF heating efficiency.

Complex 3D objects such as a prosthetic knee implant can be heated to therapeutic temperatures with AMF, but surface uniformity of heating needs to be considered both for antibacterial efficacy and to reduce tissue damage. One limitation of this study is that the *in vitro* AMF exposures were only 20 W, which resulted in a relatively slow increase in washer temperature. While the principle of bacterial destruction was still demonstrated, an equivalent surface temperature could be achieved in much shorter periods as the AMF power is increased, as shown in Fig. 7d.We are currently exploring coil arrays as a means of achieving rapid and uniform heating to increase efficacy.

This unique combination of electromagnetic and acoustic technology represents a powerful non-invasive method for targeting biofilm on the surface of metal implants within the body. These experiments demonstrate that AMF exposures are effective at eradicating biofilm *in vitro* and safety is feasible based on modelling and preliminary *in vivo* experiments. We are currently developing an infection model of a metal implant to directly evaluate the effectiveness of this method.

In summary, it is conceivable that this methodology may have applications that extend well beyond the treatment of prosthetic knee implants to other types of prosthetic implants (hips, shoulders, spine, etc), orthopedic hardware (pins, rods, clamps), stents, cardiovascular implants, foreign bodies, shrapnel, and dental implants, which in combination surpass millions of implants per year across the US alone. In an era where antibiotic resistance is of paramount concern, strategies using physical energy may offer an ability to eradicate bacteria or render them more sensitive to conventional antibiotics.

### Outlook

The results described in this study support the hypothesis that AMF exposures can eradicate biofilms on metal implants, and that this concept can be scaled up to human-sized implants. Further study and development of this approach is warranted, as it could transform the management of prosthetic joint infections from several surgical procedures to a non-invasive outpatient treatment.

## Methods

### *In vitro* AMF exposure system

A solenoid coil (5 cm diameter, 4 cm length, 40 turns) was constructed to expose biofilm-coated metal washers to an AMF for *in vitro* studies. A 505 kHz alternating current at a power of 20 W was transmitted through the coil, using a function generator (33250 A, Keysight Technologies, USA) and RF amplifier (NP-2945, NP Technologies, USA) under computer control. The forward and reflected power was measured using a directional RF power meter (NRT, Rohde and Schwarz, Germany). Power transfer to the coil was achieved constructing a series LC resonant circuit (where the solenoid represented the inductor). Centrifuge tubes (50 ml, P/N 430290, Corning, NY) containing stainless steel washers (Alloy 316, 19.1 mm OD, 4.4 mm ID, P/N 91525A107, McMaster-Carr, Douglasville, GA) were inserted into the solenoid such that the washers were centered in the coil. The infected washers were exposed to an AMF from 0.5 to 15 minutes to evaluate the relationship between AMF exposure duration and bacterial survival. Experiments were also conducted to characterize the magnitude and rate of heating of the washers using a fiber-optic temperature sensor (T1, Neoptix Inc, Quebec, Canada) attached to the top surface of a metal washer with a thermally conductive epoxy (ASTA, Arctic Silver Inc, Visalia, CA). The temperature of the washer was recorded at a rate of 1 Hz during a 10 min AMF exposure at 20 W. The measurements were repeated three times to evaluate the repeatability of the heating with the solenoid.

### *In vitro* studies

#### Bacterial Strains

Biofilm was grown on stainless steel washers using the gram-negative pathogen *Pseudomonas aeruginosa* or gram-positive pathogen S*taphylococcus aureus*. *P. aeruginosa* is the most prevalent cause of PJI among gram-negative bacteria^42^, whereas among the gram-positive organisms *S. aureus* is commonly associated with prosthetic joint infections^43^. An isolated colony of *P. aeruginosa* (PA01) was inoculated into 3 ml of cation adjusted Mueller Hinton II (MH II) media (Becton Dickinson, NJ, USA), and incubated for 18 h at 37 °C, 220 RPM. A working solution was created by 1:2500 dilution in MHII, producing an approximate bacterial concentration of 5 × 10^5^ CFU/ml. A biofilm was produced by placing a washer in a 50 ml centrifuge tube along with 4 ml of the working solution, and incubated for 18 h at 37 °C, 110 RPM. For *S. aureus* (CA-MRSA USA300 strain JE2^44^, provided by Dr. Pamela Hall, University of New Mexico), Trypticase Soy Broth (TSBG, Becton Dickinson, USA) supplemented with 1% glucose (Acros, NJ, USA) was used to form a biofilm on a washer using a method based on Lee *et al*.^45^. 3 ml of TSBG was inoculated with an isolated colony of JE2 and incubated for 24 hours at 37 °C. A working solution was prepared by 1:10 dilution in TSBG. A biofilm was produced by immersing the washer with 4 ml of the working solution in a 50 ml centrifuge tube and incubating at 37 °C for 2 hours to allow adherence, and then transferred into another 50 ml centrifuge tube containing 4 ml TSBG, and incubated for 18 hours at 37 °C, 110 RPM.

#### Colony reduction studies

Washers with biofilm were transferred into a 50 ml centrifuge tube containing 4 ml DPBS, and then placed into the solenoid and exposed to a 20 W AMF. A control tube was also included in all runs that was not exposed to AMFs but went through all the transfer steps. After each exposure, centrifuge tubes were kept on ice until ready for further processing. The biofilm CFU was quantified by transferring the exposed washers into a 50 ml centrifuge tube containing 4 ml DPBS and sonicating for 10 minutes in order to dislodge bacteria into suspension. This suspension was serially diluted, and colony-forming units (CFU) were enumerated on sheep blood agar plates (Thermo Scientific, Waltham, MA)^46^. For longer AMF exposures, 100 μl of undiluted bacterial suspension was cultured on a blood agar plate to achieve a lower limit of detection (1 Log_10_). A Spearman rank correlation and 95% confidence interval was used to assess the trend between bacterial number and exposure duration. An analysis of variance (ANOVA) model was used to compare mean bacterial number among different exposure durations. A one-sided multiple comparison with exposure time = 0 s as the control and Dunnett adjustment was used. Two sets of alternative hypotheses were tested: (1) mean bacterial number reduction was more than zero, and (2) mean bacterial number reduction was more than 3 log_10_.

#### Antibiotic studies

Washers with an 18 hour PA01 biofilm were transferred into a 50 ml centrifuge tube containing 4 ml of DPBS, and received a 3 minute AMF exposure at 20 W. The washers were then incubated in MHII containing ciprofloxacin (0, 0.125, 0.25, or 0.5 ug/ml) for another 18 hours. After incubation the biofilm CFU were determined as described above. For each concentration, a washer was also incubated without any AMF exposure to serve as a control. An ANOVA model was used to compare mean changes in bacteria number among different concentrations of ciprofloxacin and between groups with and without AMF at each concentration. Tukey adjustment was applied for all pairwise comparisons.

#### Crystal violet studies to evaluate biofilm response to AMF

The impact of AMF exposures on the EPS matrix of a PA01 biofilm was evaluated using a crystal violet (CV) assay^40^. Washers with biofilm exposed to AMF were gently rinsed in 160 mM NaCl for 1 minute. The washers were then fixed in a 4 ml of 100% methanol for 15 minutes and dried overnight. Washers were transferred into 4 ml of 0.7% w/v CV stain and placed on an elliptical shaker at the lowest setting for 20 min to allow the stain to interact fully with the biofilm. Unbound CV was removed by rinsing the washers in a 4 ml solution of 160 mM NaCl. The amount of bound CV was eluted in 4 ml solution of acetic acid (Fisher Scientific, USA) for 10 min on an elliptical shaker at the highest setting. 1 ml of the solubilized CV was transferred into a plastic cuvette, and the optical density of the solution was measured at 570 nm using a spectrophotometer (Gensys20, Thermo Scientific, USA). The measured optical density was used as a surrogate measure of biofilm burden. A Spearman rank correlation and 95% confidence interval was used to assess the trend between CV and exposure duration. An ANOVA model was used to compare mean optical density among different exposure durations. A one-sided multiple comparison with exposure time = 0 s as the control and Dunnett adjustment was used.

#### Acoustic detection of boiling

An apparatus comprised of a 4-turn solenoid operating at 517 kHz and an acoustic hydrophone (SQ26–10, Cetacean Research Technology, USA) was constructed. The system could deliver AMF exposures up to 900 W to objects within the solenoid, and permitted real-time monitoring of acoustic emissions and fiberoptic temperature during AMF exposures. A tissue-mimicking phantom was fabricated according to a previously described recipe^41^, and a 4.8 mm stainless steel ball was embedded in the center of a 50 ml centrifuge tube. A hole was drilled into the center of the ball and a fiberoptic temperature sensor was epoxied in place using a thermal compound (ASTA, Arctic Silver Inc, Visalia, CA). The frequency spectrum of the acoustic emissions before and during boiling were recorded and compared with the temperature of the ball for a range of AMF powers and durations. These recordings were obtained for the phantom, and a mouse model with an embedded metal ball (described below).

### Microscopy Studies

#### Scanning Electron Microscopy (SEM)

Washers with *S. aureus* or *P. aeruginosa* biofilm were imaged with SEM to evaluate the effect of AMF exposures on the biofilm. Imaging was performed on washers receiving 1, 3, or 5 minute AMF exposures, as well as control washers with biofilm but no AMF exposure, and a blank washer with no biofilm or AMF exposure. The protocol used to grow biofilm on the washers was the same as for *in vitro* studies described above. Washers were carefully transferred from their growth media into a 4 mL solution of DPBS, and were rinsed in 4 mL of 0.1 M sodium cacodylate buffer (6131–99–3, Electron Microscopy Sciences, Hatfield, PA) three times, and fixed for 22 h in a 4 mL solution comprised of 2% glutaraldehyde (111-30-8, Electron Microscopy Sciences, Hatfield, PA), and 2% paraformaldehyde (157–8, Electron Microscopy Sciences, Hatfield, PA) in a 0.1 M sodium cacodylate buffer (pH 7.4). After three rinse steps, the samples were then fixed in 4 mL of 2% osmium tetroxide (19160, Electron Microscopy Sciences, Hatfield, PA) in 0.1 M sodium cacodylate buffer for 2 hours. The fixed washers were rinsed with 4 mL of deionized water five times and dehydrated at room temperature in seven steps by placing the washers for 5 min in 4 mL of 50, 70 (twice), 85, 95 (twice) and 100% ethanol respectively. The washers were then transferred into 4 mL of 25, 50, 75 and 100% (twice) hexamethyldisilazane (HMDS, 999-97-3, Electron Microscopy Sciences, Hatfield, PA) in ethanol respectively for 15 min. Finally, the samples were left to dry for 24 h in a fume hood. This sample preparation protocol has been described previously for SEM of biofilms^47^. The dried specimens were mounted on aluminum stubs, sputter coated with gold/palladium, and examined using the scanning electron microscope (Zeiss ΣIGMA VP Field Emission Scanning Electron Microscope, Carl Zeiss Microscopy LTD, UK).

#### Confocal microscopy

Spinning disk confocal microscopy was performed on washers infected with a Green Fluorescent Protein (GFP) expressing *Pseudomonas aeruginosa* (GFP-PAO1, provided by Dr. Joanna Goldberg at Emory University). This strain has a pSMC2 plasmid containing GFP48. GFP-PAO1 was cultured in the same manner as above for PAO1, and underwent AMF exposures of 0 (control), 1, 3, and 5 minutes. Exposed washers were fixed in a 3 ml solution of 5% glutaraldehyde (Sigma Aldrich, St. Louis, MO) at 37 °C for 30 minutes and protected from light. Next, washers were rinsed for 1 min in 3 ml of DPBS to remove excess glutaraldehyde, and were then incubated in 200 µg/ml of an Alexa Fluor 647 conjugate of concanavilin A (ConA, C21421, ThermoFisher Scientific, Waltham, MA) for 30 minutes at room temperature (protected from light) to stain the biofilm EPS. The stained washers were placed on a coverslip and slideholder, and were imaged on the confocal microscope (Ultraview, Perkin Elmer, Waltham, MA). All images were acquired using Volocity v5.4.0 (Improvision, USA). A Zeiss plan achromat 40x/1.3 N.A Oil objective (Carl Zeiss, Thornwood, NY) and a Hamamtsu EMCCD C9100 (Hamamatsu, USA) camera were used to acquire images. The confocal effect was achieved through a spinning disk giving an XY resolution of 0.207 microns. The biofilm was imaged with a 568 nm laser and the emission filter was selected to be 615 nm with a bandwidth of 70 nm. The biofilm embedded GFP-PA01 on the washer was imaged with an Argon 488 nm laser for excitation and a 527 nm emission filter with a bandwidth of 55 nm. Multiple regions of the washer were randomly selected and Z-stacks were acquired with a step size of 0.2 microns. Before image processing the z-stacks were deconvolved using Autoquant × 3 (Media Cybernetics, Rockville, MD) to improve the resolution of the images in the X, Y and Z directions. The deconvolved images were analyzed using Bitplane Imaris 8.2.1 (Bitplane USA, Concord, MA).

### Finite Element Analysis (FEA) Simulations

Electromagnetic (EM) and thermal simulations were performed using a commercial finite-element software package (COMSOL Multiphysics v5.2, COMSOL AB, Stockholm, Sweden) to study the interaction between AMF exposures and metal implants. Tables of material and physical constants used in the simulations are included as supplementary material.

#### Geometry and Materials

The cylindrical symmetry of the washer *in vitro* setup enabled the use of a 2D axisymmetric model containing the washer, PBS, plastic tube, coil, and surrounding air extending 15 cm radially from the coil. Material properties for the stainless steel washer were obtained from the manufacturer’s datasheets, while constants for the rest of the model were assigned using COMSOL’s built-in material library. For the more spatially complex knee implant heating, a 3D model was required. An explanted prosthetic knee joint obtained at our institution was 3D scanned and converted to a Non-uniform Rational B-spline (NURBS) model, and imported into COMSOL. A coil with 20 turns, 6.5 cm radius, and a 1 cm pitch was modeled, and the knee model was centered and aligned axially within the coil. A cylindrical muscle analog was placed within the coil surrounding the knee implant, and the whole model was placed in an air volume extending 20 cm radially. For simplicity, both parts of the knee implant were modeled as 316 stainless steel, with constants for the rest of the model obtained from COMSOL’s built in library, and the ITIS tissue property database^48^.

#### Physics Modeling

Electromagnetic (EM) modeling of Maxwell’s equations was implemented with the Magnetic Field module in COMSOL. The coil for the washer was modeled using the Single-Turn Coil domain, supplied with 20 W of power, and the coil for the knee implant was modeled as an impedance boundary condition with a lumped port supplied with 250 A of current. Heat transfer (HT) in the washer was modeled using the Heat Transfer in Solids Module, and the Laminar Flow Module was used to model the Navier-Stokes equation in the PBS, with all 3 modules linked using Multiphysics. HT in the knee was modeled using the Bioheat Transfer Module, and was coupled to the EM physics by assigning and scaling the calculated resistive losses in the model from the EM physics as a heat source in the HT physics. Two cases were considered for the knee heating: a continuous heating case, where 603 watts was supplied and switched off after 3 seconds, and an intermittent case, where 1809 watts was supplied for 1 second every 50 seconds, for a total of 1200 seconds.

#### Meshing and Solving

The washer model was meshed using a physics-controlled sequence with an “extremely fine” element size, for a total of 69070 triangular elements. The knee model was meshed by hand, with a boundary layer such that there were at least 4 elements within the skin depth, for a total of totaled 300282 domain elements, 39926 boundary elements, and 12059 edge elements. Both models were solved in two steps. First, a frequency-domain solver was used to solve the EM physics, at 610 kHz for the washer and 300 kHz for the knee. Then, a time-dependent solver was used to solve the HT physics, which ran for 10 minutes for the washer, 1200 seconds for the intermittent heating knee case, and 100 seconds for the continuous heating knee case. Total solution time for the washer was 3 minutes and 21 seconds on a Windows PC with an Intel i5 processer and 32 GB of RAM, and for the knee was 1 hr 15 minutes on a Windows 7 PC with a 6 core Intel i7 processor and 128 GB of RAM.

### *In vivo* Studies

A preclinical study was performed to evaluate the tissue damage produced adjacent to implants exposed to AMF, and the ability to detect acoustic emissions associated with the onset of boiling. These experiments were approved by the Institutional Animal Care and Use Committee (IACUC) at UT Southwestern Medical Center, and conformed to the National Institutes of Health’s PHS Policy on the Humane Care and Use of Laboratory Animals. Female Swiss Webster mice (30–40 g, mean = 32.5 g, n = 42) were anesthetized using an IP injection of ketamine (80 mg/kg) and xylazine (5 mg/kg), and a subcutaneous injection of Buprenorphine (slow release) (0.6 mg/kg) was administered for pain control. The fur on the thigh was shaved and depilated, and the exposed skin was sterilized with providone iodine and alcohol. An incision was made on the thigh to create a pocket in muscle deep enough to insert a 4.8 mm diameter stainless steel ball (P/N 96415K73, McMaster-Carr, Douglasville, GA). The incision was closed with coated VICRYL 5-0 absorbable suture (J391, Ethicon, Somerville, NJ), and animals were recovered for 7 days. Animals were then anesthetized with isoflurane (1–3%), secured to a platform, and inserted into a solenoid coil such that their thigh with the implanted ball was centered along the length of a custom-built solenoid coil. The coil utilized for these studies was the same as described earlier for *in vitro* boiling detection (Fig. 7a). AMF exposures were continued until boiling was detected acoustically, indicating the same final surface temperature on the ball all animals. One group of animals (n = 5) received AMF exposures with a low power (190–260 W) and long duration (220–300 s) to match the temperature history observed in the washer experiments. Another group (n = 14) received higher powers (800–900 W) which indicated boiling in a much shorter time (4–12 s), matching the intermittent high power exposures delivered in the phantom studies. Some of the animals receiving the high power AMF exposures (n = 6) were survived for an additional 7 days to observe the delayed tissue effects in the muscle. Control groups of animals were sacrificed 48 hours (n = 2), 7 days (n = 2) and 14 days (n = 2) after implantation and received sham treatments to match the exposed animals in terms of handling and insertion in the coil. The remaining animals (n = 18) were used to develop the surgical technique, calibrate powers, refine the histological processing method, or died during surgical implantation. Upon sacrifice, the leg with the implant was harvested and fixed in formalin for 5–7 days. The tissue was then transferred to a decalcifying solution (Cal-Rite, 5501, Thermo Fisher Scientific, Waltham, MA) for 14 days to decalcify the tibia & femur. Once decalcified, the ball was carefully removed from the leg (leaving behind a cavity), and the limb was processed and embedded in a paraffin block. H&E-stained tissue sections were obtained through the location where the ball was implanted to evaluate the radial extent of thermal damage around the implant, and were reviewed by a pathologist.

## Electronic supplementary material

Supplementary Information

Intermittent AMF pulse video

Single AMF pulse video
