# Supplementary material for: Employing high-frequency alternating magnetic fields for the non-invasive treatment of prosthetic joint infections
Source: Sci Rep. 2017 Aug 8;7:7520. doi: 10.1038/s41598-017-07321-6 (PMC5548742; doi:10.1038/s41598-017-07321-6)
Supplement: Supplementary file 1 — Supplementary Information [file 41598_2017_7321_MOESM1_ESM.pdf]

Manuscript Title: Employing high-frequency alternating magnetic fields for the non-invasive treatment of prosthetic joint infections

Authors: Rajiv Chopra, Sumbul Shaikh, Yonatan Chatzinoff, Imalka Munaweera, Bingbing Cheng, Seth M. Daly, Yin Xi, Chenchen Bing, Dennis Burns, David E Greenberg

Supplementary Table S1: Material & Physical Constants used in Finite Element Simulations

Material Constants for FEA Simulations		
Parameter	*Value*	*Units*
**316 Stainless Steel**		
Thermal conductivity	16.30	W/(m.K)
Electrical conductivity	1.35e6	S/m
Specific Heat Capacity	0.5	J/(g.K)
Density	8000	Kg/m^3
**Copper**		
Thermal conductivity	400	W/(m.K)
Electrical conductivity	6.00e7	S/m
Specific Heat Capacity	385	J/(kg.K)
Density	8700	Kg/m^3
**Saline**		
Thermal conductivity	piecewise	W/(m.K)
Electrical conductivity	5.50e-6	S/m
Specific Heat Capacity	piecewise	J/(kg.K)
Density	piecewise	Kg/m^3
**Plastic Tube**		
Thermal conductivity	0.18	W/(m.K)
Electrical conductivity	5.50e-6	S/m
Specific Heat Capacity	1470	J/(kg.K)
Density	1190	Kg/m^3
**Air**		
Thermal conductivity	piecewise	W/(m.K)
Electrical conductivity	1.00e-6	S/m
Specific Heat Capacity	piecewise	J/(kg.K)
Density	piecewise	Kg/m^3

**Muscle**		
Thermal conductivity	0.49	W/(m.K)
Electrical conductivity	0.60	S/m
Specific Heat Capacity	3421	J/(kg.K)
Density	1090	Kg/m^3
Perfusion	37	Ml/kg/min
Body Temperature	37	C
Blood Density	1060	Kg/m^3
Washer Simulation Parameters		
Coil Radius	0.0175	in
Coil Diameter	1	in
Coil Pitch	0.04	In
Number of Turns	37	1
Washer Height	0.04	In
Washer outer radius	0.735	In
Washer Inner Radius	0.45	in
Water Height	1.1	in
Coil Power	20	Watts
Initial Temperature	23	degC
Frequency	610	kHz
Velocity Field in Water	6.5e-5 **r +** 6.5e-5 **z**	m/s
Heat Source	mf.Qh	Watts/m^3
Boundary Temperature	19 + step(t=10)*30	C
Knee Simulation Parameters		
Coil Radius	6	cm
Frequency	500	kHz
Number of Turns	20	1
Pitch	1	Cm
Air Temperature	19	C
Excitation Current	250	Amps
